# Age of onset of episodic and chronic cluster headache – a review of a large case series from a single headache centre

**DOI:** 10.1186/s10194-016-0626-9

**Published:** 2016-04-22

**Authors:** Gian Camillo Manzoni, Arens Taga, Marco Russo, Paola Torelli

**Affiliations:** Centro Cefalee, Dipartimento di Emergenza-Urgenza e Area Medica Generale e Specialistica, Azienda Ospedaliero-Universitaria di Parma, Padiglione Barbieri 3° piano, Via Gramsci 14, 43126 Parma, Italy; Dipartimento di Medicina Clinica e Sperimentale, Università degli Studi di Parma, Parma, Italy

## Abstract

**Background:**

In the largest case series of cluster headache (CH) published in the literature, age of onset varies between 29.6 and 31.6 years. Differences in onset age based on gender and subtype diagnosis are reported, while there are only few data on patients with childhood and elderly onset. We therefore deemed it useful to review our own large case series of CH patients.

**Methods:**

The age of onset of cluster headache was investigated in a consecutive case series of 808 patients (585 men and 223 women), including 686 (503 men and 183 women) with episodic cluster headache (ECH), 103 (66 men and 37 women) with chronic cluster headache (CCH), and 19 with an indeterminate form of CH (16 men and three women).

**Results:**

The mean age of onset was 30.2 ± 13.8 years (30.1 ± 13.0 in men and 30.4 ± 15.7 in women). The women with primary CCH had a mean onset age of 42.8 ± 21.7 years, while the women with secondary CCH did not differ much from those with ECH. Distribution of the study subjects by decades of onset age showed a peak in the third decade both in men and in women, but when only CCH patients were considered it displayed a more marked bimodal pattern in women (with peaks in the second and the sixth decade) than men (with peaks in the third and the fifth decade). The clear male predominance in cases with onset in the central age groups became attenuated in the extreme age groups. In patients with onset between ≤ 15 years and ≥ 50 years, the traditional male-to-female ratio was actually inverted in CCH.

**Conclusions:**

Based on these epidemiological findings, it would be important to investigate the possible role, causative or protective, played by hormonal factors in CH pathogenesis.

## Background

In the largest case series of cluster headache (CH) published in the literature, age of onset varies between 29.6 [1], 30.7 [2] and 31.6 years [3], namely between 29.7 [4] and 31.3 years [5] in men and between 29.4 [5] and 32.8 years in women [3]. Moreover, as already reported by Horton in 1964, chronic CH (CCH) is likely to occur at a later age [6]; indeed, all subsequent literature indicates [1–3, 7, 8] a mean age of CCH onset varying between 33.4 [1] and 39.0 years [7] versus between 28.4 [7] and 30.5 years [3] for episodic CH (ECH).

As early as 1980, Kudrow reported that “peculiar to the female distribution, an increased frequency occurred between the ages of fifty and sixty years” [1]; later studies showed this to be true especially for women with CCH [2, 4].

While CH onset in the elderly is not frequent but possible [2], in children and adolescents it is so rare that the few studies published in the literature on CH in these age groups concern very small numbers of patients [9–11].

Recently, Zidverc-Trajcovic et al. [12] have considered as early onset a CH onset age under 20 years, common onset between 20 and 40 years, and late onset over 40 years. However, in nearly half of CH patients the disease sets in before 20 or after 40; so, the age limits suggested by these authors do not seem adequate to delineate atypical onset ages for CH. In order to exactly define them, a different distribution, based not on decades but on more restricted age groups, would be needed, which obviously requires very large cases series.

We therefore deemed it useful to review our own large case series of CH patients seen at the Parma Headache Centre in order to: (a) define onset age distribution by age groups; (b) evaluate possible differences in onset age based on gender and subtype diagnosis (episodic or chronic CH); (c) determine the number of cases and distribution by gender and subtype diagnosis among patients with childhood and elderly onset; and (d) detect the age limits below and above which onset ages can be considered “atypical”.

## Methods

We considered all consecutive patients referred to the Parma Headache Centre between November 1975 and November 2015 who were affected by CH and diagnosed by our team of trained neurologists.

CH diagnosis had originally been established according to the 1962 Ad Hoc Committee on Classification of Headache [13] for the pre-1988 referrals and on the International Headache Society (IHS) classifications for post-1988 referrals [14–16]. All patients were exhaustively evaluated in face-to-face interviews to obtain valid and detailed information; a neurological examination was performed and secondary headache diagnoses were excluded.

We then reviewed our patients’ clinical records – which have not changed since 1975 and have ever since reported all clinical information required for diagnosis – and revised all CH diagnoses prior to 2013 applying the current criteria of the beta version of the 3rd edition of the International Classification of Headache Disorders (ICHD-3 beta) [16]. During this diagnostic revision, 85 patients were excluded from our case series for a variety of reasons: age of onset not known (32 patients), misdiagnosis with paroxysmal hemicrania for pre-1988 cases (28 patients), and lack of sufficient clinical information for a certain diagnosis of CH (25 patients).

The overall sample comprised 808 CH patients (585 men and 223 women).

For CCH cases, we considered separately those with primary CCH, i.e. chronic ab initio, and those with secondary CCH, i.e. evolving from ECH [14, 15]; this distinction is not present in the current IHS classification [16], but we think it could be of clinical interest.

In 19 cases, we could not define a clear temporal pattern, i.e. episodic or chronic, because some of them seemed to cross each other, while others were referred to our Headache Centre during their first cluster bout and were “lost” during the subsequent follow-up.

For the determination of CH age onset, we therefore considered only those cases in which this information could be established with certainty; comparisons by gender and subtype diagnosis were performed.

In our analysis, atypical onset ages were defined by convention as those under or over which no more than 10 % of cases are represented.

Informed consent was obtained from all subjects who participated in our study.

### Statistical analysis

Student’s t-tests for independent samples were used for comparisons between means. The collected data were analyzed using SPSS, version 20.0 for Windows. We calculated two-tailed p-values and set statistical significance at p ≤ 0.05.

## Results

Our case series comprised 808 patients with CH, including 585 men (72.4) and 223 women (27.6 %). The male-to-female (M:F) ratio was 2.6:1. A total of 686 patients (503 men and 183 women) had ECH, 103 (66 men and 37 women) had CCH, and 19 had an indeterminate form of CH. The M:F ratio was 2.7:1 in ECH patients and 1.8:1 in CCH patients.

The mean age of onset in our 808 CH patients was 30.2 ± 13.8 years (range 1–75), namely 30.1 ± 13.0 years in the 585 men (range 1–75) and 30.4 ± 15.7 years (range 5–74) in the 223 women.

The mean onset ages of patients with ECH and CCH, both male and female, are reported in Table 1. Female patients were decisive in making the differences in onset ages of ECH and CCH reach statistical significance (*p* = 0.004) (Table 1). The primary form of CCH had a later onset than the secondary form, especially in women (Table 1).

**Table 1:** Age of onset of cluster headache by sex and subtypes

		Mean (yrs)			SD (yrs)		
		Males	Females	Total	Males	Females	Total
Episodic		29.7	29.0 (1)	29.5	12.9	14.2	13.3
Chronic							
	All chronic	32.0	37.2 (1)	33.9	13.4	21.1	16.6
	Primary	35.7	42.8	38.5	16.3	21.7	18.8
	Secondary	28.9	30.8	29.5	9.5	18.9	13.1
Total		30.1	30.4	30.2	13.0	15.7	13.8

(1) Student’s test: episodic cluster headache vs chronic cluster headache among females *p* = 0.004

**Fig. 1:**
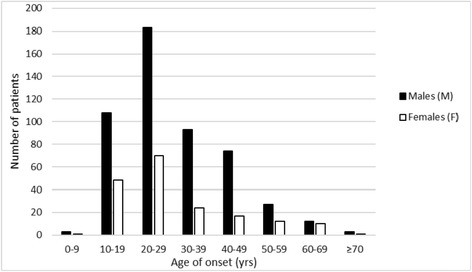
Age of onset by decades and gender (808 cluster headache cases)

Onset age distribution by decades peaked in the third decade for both sexes and showed a high number of onsets in the second and the fourth decade among men and in the second and the fifth decade among women (Fig. 1). From the sixth decade onward, the number of onsets decreased considerably in men and much less so in women; so, the M:F ratio was reduced to 1.4:1 for the 90 cases with onset at ≥ 50 years and to 1:1 for the 34 cases with onset at ≥ 60 years (Fig. 1).

**Fig. 2:**
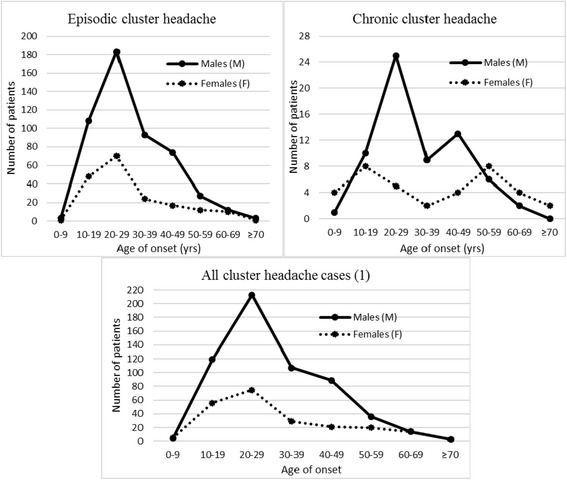
Cluster headache age of onset by gender and subtype

When only CCH cases of both sexes were considered, onset still peaked in the third decade (30 out of 103 cases), but a bimodal pattern emerged with a second peak in the fifth and the sixth decade (30 cases overall). Considering the two sexes separately, the two peaks were in the third and the fifth decade among men (25 and 13 cases out of 66, respectively), and in the second and the sixth decade among women (eight cases each out of 37) (Fig. 2). ECH did not show the same bimodal pattern as CCH, with a single peak in the third decade for both sexes.

Onset age distribution showed that there were few cases with onset in the first decade and from the sixth decade onward; by contrast, there were several cases with onset in the second decade (175, i.e. 119 men and 56 women) and also in the fifth decade (110, i.e. 89 men and 21 women).

As our case series was large enough to allow it, in order to have a more precise idea of onset age in relation to a subject’s age we carried out a distribution analysis by five-year age groups, which in our opinion was more adequate than age distribution by decades to better detect the likelihood of childhood and elderly onset.

We were therefore able to find that the high number of cases seen in the second decade was basically the result of onset in the 15–19 age group (130 of the 175 cases of the second decade), while in the 10–14 age group there was a sharp reduction (only 45 cases) (Table 2). We found a similar, though less marked pattern, in the two five-year periods of the fifth decade (70 out of 110 cases in the 40–44 age group and only 40 cases in the 45–49 age group) (Table 2).

**Table 2:** Cluster headache (CH) age of onset by age groups, gender and subtypes

	Episodic CH			Chronic CH			All CH cases (1)		
Age of onset (yrs)	Males	Females	M:F	Males	Females	M:F	Males	Females	M:F
0–4	1	0	-	0	0	-	1	0	-
5–9	2	1	2.0	1	4	0.3	3	5	0.6
10–14	26	15	1.7	2	2	1.0	28	17	1.6
15–19	82	33	2.5	8	6	1.3	91	39	2.3
20–24	106	47	2.3	11	0	-	122	47	2.6
25–29	77	23	3.3	14	5	2.8	91	28	3.3
30–34	56	12	4.7	4	0	-	62	15	4.1
35–39	37	12	3.1	5	2	2.5	45	14	3.2
40–44	49	12	4.1	7	2	3.5	56	14	4.0
45–49	25	5	5.0	6	2	3.0	33	7	4.7
50–54	17	7	2.4	5	5	1.0	23	12	1.9
55–59	10	5	2.0	1	3	0.3	13	8	1.6
60–64	6	9	0.7	1	2	0.5	7	11	0.6
65–69	6	1	6.0	1	2	0.5	7	3	2.3
70–74	2	1	2.0	0	2	-	2	3	0.7
≥75	1	0	-	0	0	-	1	0	-
Total	503	183	2.7	66	37	1.8	585	223	2.6

(1) Among all cluster headache cases, 19 cases had an indeterminate temporal pattern

**Fig. 3:**
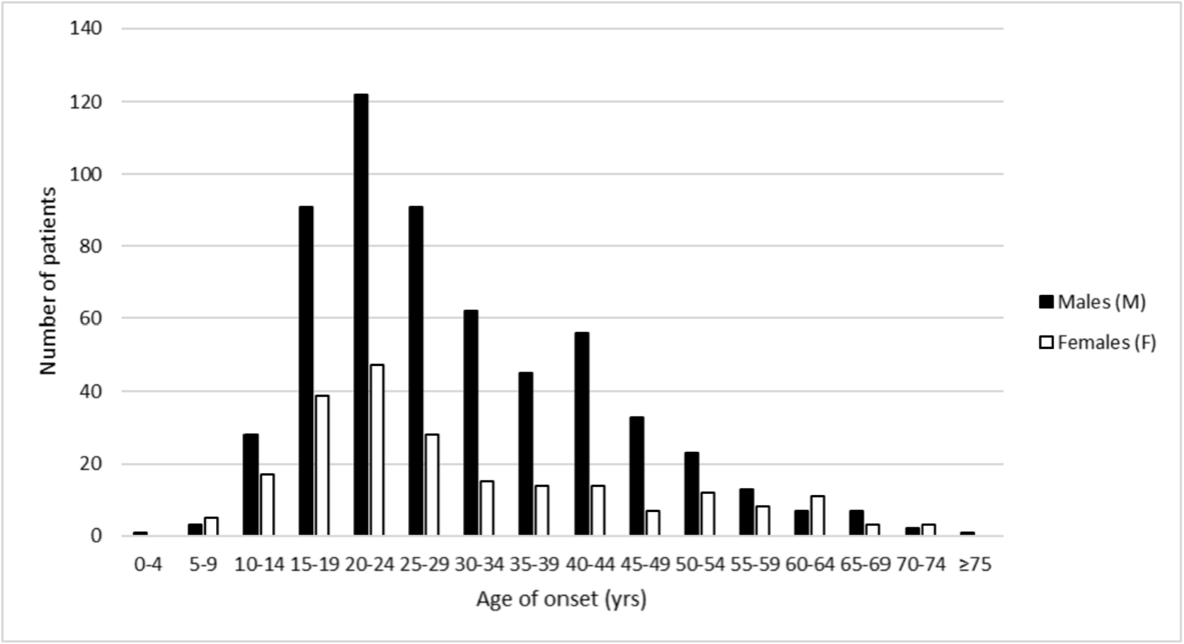
Age of onset by five-year age groups and gender (808 cluster headache cases)

Moreover, while the remarkable reduction in the number of patients with onset in the 10–14 age group became progressively more accentuated in the previous five-year periods, the decrease found in the 45–49 age group was less marked and was not followed by a similarly progressive and accentuated reduction in the next five-year periods, especially in women (Table 2 and Fig. 3).

CH set in at ≤ 14 years in 54 of our 808 patients, or 6.7 % (32/585 men, 5.5 %; 22/223 women, 9.9 %), at ≥ 50 years in 90, or 11.1 % (53/585, 9.1 %; 37/223, 16.6 %), and at ≥ 55 years in 55, or 6.8 % (30/585, 5.1 %; 25/223, 11.2 %) (Table 2).

The M:F ratio was 1.5:1 in patients with onset at ≤ 14 years, 1.4:1 in those with onset at ≥ 50 years, and 1.2:1 in those with onset at ≥ 55 years.

However, if ECH and CCH were considered separately, women were more numerous than men in these onset age groups of CCH; indeed, the M:F ratio in patients with onset at ≤ 14 years was 1.8:1 in ECH and 0.5:1 in CCH; in patients with onset at ≥ 50 years it was 1.8:1 in ECH and 0.6:1 in CCH (Table 2).

When considering atypical onset age as previously defined, it appeared to differ in the two sexes: it varied between ≤ 15 years (52/585 cases, 8.9 %) and ≥ 50 years (53/585, 9.1 %) for CH males and between ≤ 14 years (22/223 cased, 9.9) and ≥ 58 years (21/223, 9.4 %) for CH females (Table 3).

**Table 3:** Cluster headache age of onset divided by year (<20 and >45 years) and gender

Age of onset (yrs)	Males	Females	Total (1)	Age of onset (yrs)	Males	Females	Total
1	1	0	1	46	4	2	6
5	0	1	1	47	4	1	5
6	1	1	2	48	9	1	10
7	0	1	1	49	6	2	8
8	2	1	3	50	6 (2↓)	2	8
9	0	1	1	51	4	3	7
10	5	3	8	52	6	0	6 (2↓)
11	1	3	4	53	3	5	8
12	3	2	5	54	4	2	6
13	7	5	12	55	1	0	1
14	12	4 (2↑)	16	56	2	1	3
15	20(2↑)	5	25 (2↑)	57	4	3	7
16	12	3	15	58	3	3 (2↓)	6
17	19	10	29	59	3	1	4
18	20	11	31	60	0	3	3
19	20	10	30	61	0	3	3
(1) Among all cluster headache cases, 19 cases had an indeterminate temporal pattern.				62	1	2	3
				63	2	3	5
(2) 10 % of cases cut-off.				64	4	0	4
				65	0	2	2
				66	4	0	4
				67	2	1	3
				68	1	0	1
				71	1	1	2
				72	1	1	2
				74	0	1	1
				75	1	0	1

## Discussion and conclusions

Confirming what has already been reported by several authors [1–8], the review of our case series of CH patients shows that the mean age of onset is around 30 years. If we consider ECH and CCH separately, we can see that the mean onset age is older (33.9 ± 16.6 years) in the latter, but this is only due to the figure (38.5 ± 18.8 years) for the CCH subtype that until the 2004 international headache classification [15] was called primary CCH, i.e. chronic ab initio; by contrast, the onset age of secondary CCH, i.e. CH that has become chronic only after several years with a periodic time pattern, is entirely comparable to that of ECH (29.5 ± 13.1 year and 29.5 ± 13.3 years). In women, this difference is even more marked (42.8 ± 21.7 years for primary CCH, 30.8 ± 18.9 years for secondary CCH, and 29.0 ± 14.2 years for ECH).

The analysis of onset age distribution by age groups confirms the presence of a peak in the third decade and of a bimodal pattern only in women with a further peak at a more advanced age [1, 2, 4, 7, 17].

In addition to confirming some observations already reported in the literature about age of CH onset, with this study we were able to acquire new knowledge from our very large case series (808 patients). The only case series larger than ours was that of Rozen and Fishman in the US [17], which consisted of 1,134 patients; these, however, were recruited in the Internet and were not seen personally as were ours.

Our data show that the previously reported bimodal pattern of CH onset age in women can be observed only in CCH, with two peaks in the second and the sixth decade, respectively (Fig. 2). In CCH males too, there is a bimodal pattern, though less marked than in females, with a peak in the third decade and another one, less marked, in the fifth decade (Fig. 2).

Another very interesting finding that emerged from the review of our cases series is that the male predominance in CH is more marked in patients with onset in the more central age groups and tends to decrease in the extreme age groups. When considering CCH separately for the two sexes, we can even see that there is a female predominance both in cases with childhood onset and in cases with onset at ≥ 50 years. These are mostly primary CCH cases.

The onset age distribution by five-year age groups, the attempt to detect “atypical” onset ages and the analysis of the M:F ratio trends in relation to the different onset ages enables interesting conclusions to be drawn about women with CH. In particular, the likelihood that a woman may develop CH appears the same as, if not higher than for CCH, that of a man in pre-adolescence and from age 50 onward. This finding could be a stimulus for further studies aimed at investigating the possible role – causative or maybe protective – played by hormonal factors in CH pathogenesis.

Our study has methodological strengths and limitations that need to be considered.

Among the former are: (1) the case series that we report is very large and includes patients with CH diagnosis according to the ICHD-3 beta criteria; (2) the diagnosis was made by face-to-face examination, and secondary causes were excluded; (3) all reported cases were personally seen by the author himself (GCM).

Among the latter are: (1) the breakdown of our case series by diagnosis, sex and age group has led to small-sized subgroups, especially the subgroup of CCH women; (2) for that reason, we were not able to perform a statistical analysis to determine the significance of certain findings, in particular the bimodal pattern of onset ages in CCH women and the relatively higher proportion of women than men with onset in the extreme age groups, especially for CCH.

It would be interesting to check our findings against larger case series, particularly for CCH women.
